# Comparison of permeatal medial placement of graft without raising the tympano-meatal flaps to conventional methods of myringoplasty: An experience at tertiary care hospital in Pakistan

**DOI:** 10.12669/pjms.324.9497

**Published:** 2016

**Authors:** Saleem Asif Niazi, Zaheer Ul Hassan, Khaula Atif, Saeed Ullah

**Affiliations:** 1 Dr. Saleem Asif Niazi, MBBS, MCPS, FCPS Department of ENT, Combined Military Hospital, Peshawar, Pakistan; 2 Dr. Zaheer Ul Hassan, MBBS, FCPS Department of ENT, Combined Military Hospital, Peshawar, Pakistan; 3 Dr. Khaula Atif, MBBS, MCPS(Family-Medicine), DPH, DMA. Department of General Administration, Combined Military Hospital, Peshawar, Pakistan; 4 Dr. Saeed Ullah, MBBS, FCPS Department of ENT, Combined Military Hospital, Quetta, Baluchistan, Pakistan

**Keywords:** Myringoplasty, Permeatal, End aural/Post aural

## Abstract

**Objective:**

To compare the results of permeatal approach without raising the tympano-meatal flap to end-aural or post-aural approach in myringoplasty.

**Methods:**

This Quasi-experimental study was carried out in CMH (Combined Military Hospital) Peshawar, from August 2006 to July 2013. Three hundred fifty patients of chronic suppurative otitis media (CSOM) with dry central; small, medium and large perforations were selected. They were divided into two groups depending upon the type of approach. In Group-A (n-200); permeatal approach without raising tympano-meatal flap was used; while in Group-B (n-150) end-aural or post-aural approach was used. Subjects were followed up for two years; graft take was checked regularly by examinations of ear under microscope. Data was collected on structured Performa and analysed by SPSS-17.

**Results:**

Male and female were 74% and 26% respectively; Age ranged from 15 to 46 Years. There was no significant difference in the graft success at the end of two years in Group-A(80%) and Group-B(85%) (p-0.261). Type of approach had a significant impact on duration of surgery(p<0.001) and post-operative recovery time(p<0.001).

**Conclusion:**

The permeatal approach and end-aural/post-aural approach had almost equal graft success rates, but former is more useful as it causes lesser morbidity, decreased post-operative hospital stay and reduced operative time. It is under-utilized and should be employed more frequently.

## INTRODUCTION

Chronic otitis media is a common disorder around the world.^1^ It is more common in the developing countries including Pakistan because of the low socioeconomic condition of people in these countries.^2^-^4^

Chronic discharging ears can be grafted by different tissues once dry for at least 4-6 weeks; preferably by temporalis fascia. This surgery is called myringoplasty, which helps the patient by stopping the ear discharge and improved hearing. Moreover, it improves the quality of life like swimming and bathing.^5^ Depending on the set-up and surgical techniques; the success rate of myringoplasty ranges from 70% to 90%. It can be performed by post-aural, end-aural or permeatal routes. Depending upon the site and size of perforation any of the routes can be adopted.

Permeatal/trans-canal approach is commonly used in wide external auditory meatus with small to medium sized perforations. Tympano-meatal flap is raised and the graft is underlayed medial to the tympanic membrane. In some centers, the middle ear is filled with gel-foam and the graft is placed medial to the tympanic membrane without raising the tympano-meatal flap.^6^ Although this technique is difficult especially because space for maneuverability in the external auditory meatus is less, nevertheless it causes lesser morbidity shorter postoperative hospital stay, and better cosmetic outcome as the scar is small and that too in the hairlines, therefore practically/cosmetically no scar is visible.

The idea behind this study was to compare the success rates of Permeatal underlay myringoplasty (pop in technique) with end aural/post-aural technique for small, medium or large sized central tympanic membrane perforations along with comparison of other operative and postoperative parameters.

## METHODS

A quasi-experimental study was carried out in CMH Peshawar from August 2006 to July 2013. Commencement of study was preceded by formal approval from ethical committee of the hospital and written informed consentwas taken from patients. Data was collected on structured performa endorsing demographic details along with type and outcomes of surgery.

Adults with tubo-tympanic CSOM, having central perforation (small/medium/large) in the tympanic membrane which was dry for at least 4-6 weeks were included in the study. Exclusion criteria were total or subtotal perforations, sensori-neural hearing loss, patients having malformations of ears like too small external auditory meatus, or having foci of infection like deflected nasal septum (DNS), chronic tonsillitis. Those with systemic illnesses like diabetes or hypertension were also excluded. Sample of 350 was selected through convenient sampling.

Preoperatively patients were admitted and examined in detail and under microscope. Perforations were confirmed and drawn on patients’ charts. All relevant investigations for general anesthesia were done. Audiological investigation included pure-tone-audiogram (PTA). The cases were randomized by card method irrespective of gender, age and size of the tympanic membrane perforation into two groups on the basis of type of surgery performed. Group-A(n-200) included patients operated by permeatal underlay technique while Group-B(n-150) included those operated by end-aural/post-aural approach. The cases were operated upon by same surgeon under general anaesthesia using temporalis fascia graft. Post operatively, all patients were given I/V antibiotics for at least 04 days.

On 7^th^ post-op day, examination under microscope (EUM) of ears for graft take was done followed by pure-tone-audiometery(PTA) in 6^th^ week to assess improvement in hearing. They were then regularly followed up for two years in OPD.

Data was analyzed via SPSS-17. Graft success was the main dependent variable while gender, age, type of surgery, duration of surgery and post-operative hospital stay were independent variables. Quantitative variables expressed as mean±SD (standard-deviation), qualitative as percentage/frequencies. Cross-tabulation was done via chi-square to generate p-value; latter less than 0.05 was taken as significant.

## RESULTS

Out of 350 patients, 74% were males(n-259) and 26% were females(n-91), (male:female=2.8:1). Age was 29.23±7.64, ranging from 15-46 years. Size of tympanic membrane perforations operated were small 19%(n-67), medium 55%(n-194) and large 25.3%(n-89). After two years of follow up graft succeeded in 82%(n287).

**Fig.1:**
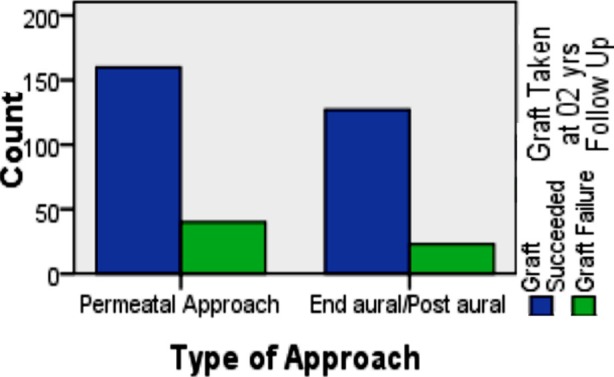
Comparison between Type of Approach & Graft Success on 2 Years’ follow-up.

Among Group-A 80% of patients had improved hearing with graft take compared to 85% in Group-B, depicting no significant difference in results(*p*-0.163)(Fig.1). In Group-B end-aural approach was used in 115(76.6%) and post-aural in 35(23.4%). Duration of procedure in Group-A was less than 45 minutes, significantly lesser than 90 minutes or more of Group-B(*p*<0.001). Postoperative recovery time in Group-A was 7 days; significantly shorter than 15 days of Group-B(*p*<0.001)(Table-I). There was no significant impact of type of approach (*p*-0.261), post-operative recovery time (p-0.261) and duration of surgery(p-0.261) on graft success after two years.

**Table-I:** The comparison between two groups of patients.

Ser	Variable	Group-A	Group-B
1.	Type of Surgery	Permeatal Approach	End-Aural/Post-Aural Approach
2.	Duration of Surgery	<45 min	>90 min
3.	Post-operative Recovery Time	7 Days	15 Days
4.	Graft Success after 2 Years	80%	85%

## DISCUSSION

The history of myringoplasty and tympanoplasty type-I dates back to its first controversial description in 1878 followed by its actual foundation in 1952,^7^ mostly German participated in its development, followed by classification of middle ear repair by Horst Wullsteinin 1958.^8^

Type I Tympanoplasty also called as myringoplasty only tympanic membrane is perforated and it is repaired. In Type-II Tympanoplasty, handle of maleus is also damaged and the graft is placed over the damaged ossicle. In type-III the malleus and incus are damaged and the graft is placed directly to the head of stapes. In type-IV the stapes crura are damaged as well and the graft is placed over the mobile stapes footplate while in type-V stapes footplate is fixed which is repaired.

Myringoplasty is a well known procedure but surgeons are determined to improve it further in terms of reduced operative time, postoperative recovery time and to make it more malleable for the patient. Endoscopic myringoplasty was also introduced for this endeavour.^9^ Objectives of myringoplasty are to repair the ear-drum and to improve hearing standards.^10^

Myringoplasty is performed for a perforated ear-drum which could be due to chronic otitis media or trauma to the tympanic membrane which had not healed in three months. Patients who are in water-sports who develop recurrent middle ear infections are candidates for myringoplasty. Similarly recurrent episodes of middle ear infections, progressive hearing loss due to middle ear infection or inability to take bath properly are indications of myringoplasty.

Tympanoplasty is performed mainly by three approaches, post-aural approach is an incision behind the post-auricular groove, end-aural approach is an incision anteriorly and permeatal approach is through the canal.^11^ Graft can be a fascia, a cartilage, muscle or fat. Most commonly temporalis fascia is taken as the first choice but in small perforations fat from tragus can be used. Permeatal surgery takes half to one hour and end-aural or post-aural surgery normally takes 2½ to three hours. The success rates are from 85% to 95%. In our setup patients are admitted and kept on I/V antibiotics for 4-5 days post-operatively.

Graft can be placed either lateral to the tympanic membrane called “on lay technique” or medial to the tympanic membrane called “underlay technique”. In underlay technique a tympano-meatal flap is created and is raised up to the annulus, graft is then pushed under this flap to close the perforation and secured by putting gel foam in the middle ear.^12^ Graft can be placed either lateral or medial to the handle of maleus making no difference.^13^

Lateral grafting means is freshening the outer side of tympanic membrane and placing the graft over it. Graft materials can even be allo-grafts but preferred materials are temporalis fascia, small cartilages or fat plugs.^14^ In this study we used a technique not commonly mentioned in textbooks. Initially we thought that we are experimenting a new technique but detailed literature review revealed some researchers have used this technique to small size perforations with wide meatus whereas we selected small, medium and large perforations with larger meatus size i.e. where a medium size ear speculum could be used. We faced problems in watching the complete margins of large perforations especially anteriorly but this problem was overcome by tilting the microscope. Now trans-meatal endoscopic procedures have overcome this problem.^15^ In order to see the anterior edge of tymanic membrane perforations a combination of microscope with endoscope were also used.^15^ The procedure adapted in our study was injecting local anesthetic agent just lateral to the bony cartilaginous junction at 3, 6, 9, and 12 o’clock positions aiming to get a clear field in case our instruments touched the external auditory meatus. The margins of perforation were freshened, mucosa of middle ear was separated from tympanic membrane by curved needle, an incision made superior to the pinna just above the hairline and the graft was excised. The middle ear was filled with Gel-foam and the graft of suitable size was placed medial to the tympanic membrane through the perforation. The graft was stabilized by putting gel foam over the graft thus sandwiching the graft in gel foam. This technique is adapted endoscopically but only for traumatic perforations.^16^

In some studies a Steri-Strips is placed over the perforation and tissue graft to stabilize the graft. Some surgeons used fat plug to close the perforation.^17^ Perforation smaller than 5% of the tympanic membrane are being closed by paper-patch technique or a piece of gel-foam is plugged in the perforation.^18^,^19^ Shortest and least morbid technique with favorable results could be seen with hyaluronic-acid fat graft myringoplasty but this material is not easily available in our setup.^20^

In our view although Permeatal underlay technique without raising the tympano-meatal flap is difficult because of less space of maneuverability in the external auditory meatus. Never the less it causes lesser morbidity, shorter postoperative hospital stay and cosmetically it is a much better technique as it leaves only small scar in hairline (leaving practically no scar). Above all, in case of procedure failure, there is a valid option available to perform conventional surgery by raising the tympano-meatal flap. Patients always prefer for an easy way out, cosmesis, quick recovery and reduced hospital stay.

This study aimed to analyze factors which could offer reduced operative time render better results of surgery thus making patients more comfortable postoperatively followed by better cosmesis and hospital stay. The perforations in our study were small to large and they were randomly placed in the two groups. The results for large perforations in permeatal approach were compatible to those of end-aural and post-aural approaches. The approach does not affect the outcome of myringoplasties provided the basics are met. The results of our study are coinciding with a study conducted in Netherland in 2009.^21^ Occam’s Razor suggested one should opt for the procedure which is simpler, fast and has lower morbidity.^22^

This study has several limitations. The sample size was small, bigger cohort could have rendered varied results, but that could turn out to be less cost-effective. The sample size was still sufficient enough to establish generalized results for local population of concerned area. Nevertheless, it is a unique study as no such study has yet been carried out in this region on similar patients. This research can assist ENT specialists to adopt better approach for surgery to generate enhanced results.
